# Serotonin Receptor 5-HT3A Affects Development of Bladder Innervation and Urinary Bladder Function

**DOI:** 10.3389/fnins.2017.00690

**Published:** 2017-12-12

**Authors:** K. Elaine Ritter, Zunyi Wang, Chad M. Vezina, Dale E. Bjorling, E. Michelle Southard-Smith

**Affiliations:** ^1^Division of Genetic Medicine, Department of Medicine, Vanderbilt University School of Medicine, Vanderbilt University, Nashville, TN, United States; ^2^Department of Surgical Sciences, School of Veterinary Medicine, University of Wisconsin-Madison, Madison, WI, United States; ^3^Department of Comparative Biosciences, University of Wisconsin-Madison, Madison, WI, United States

**Keywords:** lower urinary tract, autonomic nervous system, sensory nervous system, neural crest, Htr3a, cystometry, void spot assay, pelvic ganglia

## Abstract

The autonomic and sensory nervous systems are required for proper function of all visceral organs, including the lower urinary tract (LUT). Despite the wide prevalence of bladder dysfunction, effective treatment options remain limited. Pelvic innervation regenerative strategies are promising, but surprisingly little is known about the molecular factors driving the development of bladder innervation. Given prior evidence that serotonin receptor 5-HT3A is expressed early in LUT development and is an important mediator of adult bladder function, we sought to determine if 5-HT3A is required for the development of autonomic innervation of the bladder. We found that 5-HT3A is expressed early in fetal mouse pelvic ganglia and is maintained through adulthood. *Htr3a* knockout male mice, but not females, exhibit increased urinary voiding frequency compared to wild type littermates. Analysis of LUT function via anesthetized cystometry revealed decreased voiding efficiency in male *Htr3a* mutants. *Htr3a*^−/−^ mutant animals exhibit a transient disturbance of autonomic neuronal subtype markers (tyrosine hydroxylase and choline acetyl transferase) within the fetal pelvic ganglia, although the imbalance of neuronal subtype markers assayed is no longer apparent in adulthood. Loss of 5-HT3A activity results in a higher density of autonomic and sensory neuronal fibers supplying bladder smooth muscle in both fetal and adult mice. Collectively, our findings highlight 5-HT3A as a critical component in the autonomic control of micturition and identify a novel role for this serotonin receptor in peripheral nervous system development.

## Introduction

The lower urinary tract (LUT, bladder and urethra) is responsible for the storage and timely elimination of urine and is controlled by autonomic, sensory, and central motor innervation. The autonomic components are comprised of the pelvic ganglia (PG), which harbor both sympathetic and parasympathetic neurons (Wanigasekara et al., 2003). The coordination of these neural components allows the bladder smooth muscle to relax as it fills with urine while the internal urethral sphincter contracts to prevent urine leakage, and vice versa when the micturition reflex is initiated (de Groat et al., 2015). When any of these neural components do not function properly, urinary incontinence or retention can occur and severely diminish quality of life. The nerves supplying bladder innervation are prone to damage in instances of spinal cord injury and during surgical procedures in the lower abdomen (Zullo et al., 2003; Wyndaele, 2016). Additionally, LUT dysfunction can arise during the course of natural aging (Wagg et al., 2007). Despite the widespread prevalence of these conditions, treatment options remain limited. Regenerative strategies aimed at restoring LUT innervation are promising (Kim et al., 2013), and such efforts require a deeper understanding of the molecular processes governing the development of LUT innervation (Keast et al., 2015).

In an effort to characterize patterns of gene expression in the development of LUT sensory innervation, we previously examined expression of the serotonin receptor 5-HT3A (encoded by the *Htr3a* gene) in developing dorsal root ganglia (Ritter and Southard-Smith, 2017). In these analyses we also observed pronounced expression of *Htr3a* in fetal mouse pelvic ganglia that supply autonomic innervation to the bladder. This finding is of particular interest in the context of a previous study reporting severe voiding dysfunction in *Htr3a*^*vs*/*vs*^ mice that express a constitutively active form of 5-HT3A (Bhattacharya et al., 2004). However, the role of 5-HT3A signaling in the development of LUT autonomic innervation has not been previously examined in detail.

Given the evidence of 5-HT3A receptor activity in adult LUT function and expression of this receptor early in fetal LUT development, we tested the hypothesis that 5-HT3A is required for the development and function of bladder innervation. Using the *Htr3a*^−/−^ knockout line, we demonstrate here that 5-HT3A is required for normal micturition in male mice. Conducting the spontaneous void spot assay in pre-pubescent *Htr3a* mutant mice revealed aberrant urinary voiding in male, but not female, knockout animals. Anesthetized bladder cystometry revealed that in males, the loss of 5-HT3A receptor activity diminishes bladder voiding efficiency. At the cellular level we found that 5-HT3A is required for the maturation of autonomic and sensory neuronal fibers innervating bladder smooth muscle. Collectively our results demonstrate a novel role for 5-HT3A in peripheral nervous system development and further highlight this receptor as a critical signaling component in the regulation of micturition.

## Materials and methods

### Animals

All experimental protocols were approved by the Institutional Animal Care and Use Committee (IACUC) at Vanderbilt University and University of Wisconsin-Madison. Tg(*Htr3a*-EGFP)DH30Gsat/Mmnc (RRID: MMRRC_000273-UNC) transgenic mice were obtained from the Mutant Mouse Resource & Research Center and maintained as heterozygotes on an outbred Swiss Webster background. Tg(*ChAT*-EGFP)GH293Gsat/Mmucd (hereafter *ChAT*-EGFP) transgenic reporter mice were obtained from the Mutant Mouse Resource and Research Center and maintained as homozygotes on a C57BL6/J background. B6.129X1-*Htr3a*^*tm*1*Jul*^/J (RRID: IMSR_JAX:005251; hereafter referred to as *Htr3a*^−/−^) mice were acquired from The Jackson Laboratory and maintained on a C57BL6/J background. To generate *ChAT-*EGFP; *Htr3a-*KO animals for pelvic ganglia gene expression studies, *ChAT*-EGFP/EGFP homozygotes were bred to *Htr3a*^−/−^ mice to generate *ChAT-*EGFP/+; *Htr3a*^+/−^ animals. These progeny were then mated with *Htr3a*^+/−^ mice to produce *ChAT*-EGFP/+; *Htr3a*^+/+^, *Htr3a*^+/−^, and *Htr3a*^−/−^ animals. This breeding strategy was adopted to avoid comparing expression patterns of heterozygous vs. homozygous expression of the *ChAT-*EGFP transgene. All mice were housed in a modified barrier facility at approximately 25°C and 25–40% humidity. Mice were maintained on a 14-h on, 10-h off light cycle and housed in rectangular polypropylene micro-isolator cages (Lab Products Inc., #10025, 29.2 × 19.0 × 12.7 cm) and provided standard diet (Purina PicoLab Laboratory Rodent Diet #5L0D) and water *ad libitum*. Males and females were paired for timed matings to obtain fetal tissues at specific developmental stages; the morning of observation of a seminal plug was designated as 0.5 days post coitus (dpc).

### Tissue collection

Male and female mouse fetuses were dissected in ice-cold 1X Phosphate Buffered Saline (PBS) and fixed in 10% Neutral Buffered Formalin (NBF, Sigma Aldrich HT501128) overnight at 4°C. Following fixation, tissues were washed three times in 1XPBS for 15 min each and then finally washed for 1 h. Tissues were then infiltrated with 30% sucrose in 1XPBS and stored in the same solution at 4°C until the day of embedding and cryo-sectioning. To obtain pelvic ganglia and bladders at adult stages, mice were anesthetized by isoflurane inhalation and euthanized by cervical dislocation. A lower abdominal incision was made to reveal the LUT and the pelvic ganglia were carefully teased away from the neck of the bladder and pinned out flat on petri dishes lined with Sylgard (Ellsworth Adhesives, #4019862). The pelvic ganglia were fixed in 10% NBF overnight at room temperature while gently shaking. Bladders were removed by cutting the proximal urethra and the ureters. To remove urine and allow bladder relaxation so that smooth muscle was not stretched thin, bladders were flushed with 1XPBS and subsequently emptied using a needle and syringe before fixation in 10% NBF overnight at 4°C. Bladders and pelvic ganglia were washed three times in 1XPBS for 15 min each, followed by 1 h of washing in 1XPBS and infiltrated with 30% sucrose at 4°C.

### Immunohistochemistry

#### Cryo-sections

Fixed tissues for cryo-sectioning were embedded in Tissue Freezing Medium (TFM, General Data, #TFM) and immediately sectioned with a Leica Cryostat (CM1900-UV). Sagittal sections of 20 μm thickness were cut and mounted onto 3-APES treated slides (Sigma Aldrich, A3648) and dried for 30 min at 37°C on a slide warmer. Slides were then submerged in 1XPBS-0.3% Triton X-100 to dissolve residual TFM for 5 min. Blocking solution (1XPBS-0.3% Triton X-100, 10% Bovine Serum Albumin (Sigma Aldrich, A2153), 5% Normal Donkey Serum (Jackson ImmunoResearch, 017-000-121), 0.45 μm sterile filtered) was applied to sections for at least 30 min at room temperature. Sections were then incubated in primary antibodies diluted in blocking solution overnight at 4°C. Primary antibody information can be found in Table 1. On the following day, sections were rinsed with sterile 1XPBS and incubated in secondary antibodies diluted in blocking solution for 1 h at room temperature. Secondary antibody information can be found in Table 2. After rinsing with sterile 1XPBS, sections were incubated in 0.5 mM cupric sulfate in 50 mM ammonium acetate buffer, pH 5.0, for 10 min at room temperature to quench autofluorescence (Potter et al., 2012). Sterile water was used to quench the cupric sulfate reaction. Slides were finally mounted with AquaPolyMount (PolySciences, Inc., 18606) and coverslipped.

**Table 1:** Primary antibodies used in immunohistochemistry experiments.

**Antibody**	**Host**	**Vendor**	**RRID**	**Dilution**
Tyrosine Hydroxylase (TH)	Sheep	Millipore, #AB1542	AB_90755	1:1,000
Vesicular Acetylcholine Transporter (vAChT)	Rabbit	Synaptic Systems, #139-103	AB_887864	1:1,000
Calcitonin Gene Related Peptide (CGRP)	Rabbit	Sigma Aldrich, #C8198	AB_259091	1:1,000
Neurofilament 200 (NF200)	Rabbit	Sigma Aldrich, #N4142	AB_477272	1:500
β-III Tubulin (Tuj1)	Rabbit	Covance, #PRB-435P	AB_291637	1:10,000
Hu C/D	Human	Gift of Vanda Lennon, Mayo Clinic	N/A	1:10,000

**Table 2:** Secondary antibodies used in immunohistochemistry experiments.

**Antibody**	**Vendor**	**RRID**	**Dilution**
Donkey anti-Rabbit Cy3	Jackson ImmunoResearch, #711-165-152	AB_2307443	1:1,000
Donkey anti-Rabbit AlexaFluor 647	Jackson ImmunoResearch, #711-605-152	AB_2492288	1:250
Donkey anti-Rabbit AlexaFluor 488	Jackson ImmunoResearch, #711-545-152	AB_2313584	1:400
Donkey anti-Sheep Cy3	Jackson ImmunoResearch, #713-165-147	AB_2340728	1:1,000
Donkey anti-Human AlexaFluor 647	Jackson ImmunoResearch, #709-605-149	AB_2340578	1:200

#### Whole-mount pelvic ganglia

Following fixation and rinsing, pelvic ganglia were blocked at least 24 h at 4°C in the same blocking solution used for cryo-sections. Tissues were then incubated in primary antibody dilutions for 48 h at 4°C in a 24-well tissue culture plate and rinsed three times, 20 min each, in sterile 1XPBS. Pelvic ganglia were then incubated in secondary antibody dilutions for 1 h at room temperature while rocking. Tissues were rinsed three times, 20 min each, in sterile 1XPBS and then incubated in 0.5 mM cupric sulfate in 50 mM ammonium acetate buffer, pH 5.0, for 10 min at room temperature to quench autofluorescence. The cupric sulfate quenching reaction was quenched by transferring the tissues to sterile water. Tissues were mounted onto slides with AquaPolyMount and coverslipped and allowed to dry before imaging. Confocal microscopy of immunohistochemical experiments was conducted using a Zeiss LSM 510-Meta confocal microscope, equipped with a 20x/0.75 plan-apochromat objective.

### Spontaneous void spot assay

Spontaneous void spots assays (VSA) were conducted as published (Keil et al., 2016), but with modifications as described here. Mice of the same sex were housed in groups of five animals or fewer per cage. Whatman filter paper (3 mm, chromatography grade, GE Healthcare #3030-347) was cut into sheets to precisely fit the bottom of a standard mouse cage (see section Animals in Materials and Methods) and autoclaved in foil-wrapped packets. The assay was conducted at the same time each day (12:00–1:00 p.m.) to avoid circadian variability in micturition behavior. To conduct the assay, each mouse was placed in its own void assay cage (a cage identical to the animals' home cage, with no food, water, or bedding) with a piece of autoclaved Whatman filter paper on the floor of the cage. The void assay cages were then returned to the cage rack and left undisturbed for the duration of the assay (1 h). Voiding was assessed for 1 h to minimize stress and anxiety from prolonged food and water deprivation. At the end of the hour, the mice were carefully lifted out of the trial cages and returned to their home cages. Prior to the VSA trial days, 5 days of assay acclimation that entailed placing the animals in individual cages with Whatman paper were conducted to minimize the effects of novel environment (i.e., the assay cage and Whatman paper) on micturition behavior. Following acclimation and 2 days of rest, three consecutive trials were carried out. For each acclimation and trial, the same void assay cage was used for each animal. Acclimation and testing took place while the animals were 4–5 weeks old to minimize the effects of sex hormones and estrous cycling on micturition behavior.

The soiled sheets of Whatman paper were stained with Ninhydrin (2% w/v dilution in 190-proof ethanol, Sigma Aldrich, 151173) and allowed to air-dry overnight. Dried Ninhydrin-stained sheets were scanned to a computer as TIF files. Urine void spots were identified by their purple coloration due to Ninhydrin staining and round shape; non-urine marks were identified as either fecal material (brown spots/smudges) or paw prints (purple paw-print shapes). These non-urine artifacts were erased in Adobe Photoshop prior to analysis. The particle analysis tool in ImageJ (http://imagej.nih.gov/ij/) was used to measure the total number of void spots >0.02 cm^2^ and the surface area of each individual void spot. Staining with Ninhydrin allows clear visualization of the borders of overlapping voids (Supplementary Figure 1). Overlapping void spots were manually counted by drawing a line on the border between overlapping spots and using the freehand selection tool to count them separately. These measurements were then incorporated into the analysis. The total number of individual voids and the combined area of total voids were averaged over the 3 days of trial.

### Anesthetized bladder cystometry

Cystometric recordings were made in mice under urethane anesthesia using established methods (Uvin et al., 2012; Boudes et al., 2013; Bjorling et al., 2015; Roman et al., 2016). Briefly, mice were weighed and anesthetized with urethane (1.43 g/kg, subcutaneous injection). Thirty minutes later, the bladder was carefully exposed through a lower midline abdominal incision. A PE 50 cannula with the end flared was inserted into the dome of the bladder and secured with a 6.0 silk purse string suture. Muscle and skin layers were then closed separately with a 5.0 silk suture. One hour after cannula implantation, the distal part of the cannula was connected to a physiological pressure transducer (Memscap AS, Norway) and an infusion pump (Harvard Apparatus, Holliston, MA) via a 3-way stopcock. Room temperature saline was infused at a rate of 0.8 mL/h for 60–90 min to stabilize voiding patterns and elicit repetitive micturition cycles. Intravesical pressure was recorded continuously using a PowerLab data collection system (ADInstruments, Colorado Springs, CO) connected to a PC computer. The voided volume of saline was quantified by collecting voided saline on small filter papers weighed before and after voiding. Additionally, void volume was simultaneously measured using a force transducer (Model FT03, Grass Instruments) placed underneath the distal tip of the urethra to collect voided infusate. The force transducer was calibrated using known volumes of infusate to generate a pressure-volume curve. At least six reproducible micturition cycles were recorded and used to quantify multiple parameters. Specifically, intercontractile interval (ICI, time between micturition events), maximal voiding pressure (VP), bladder capacity (BC, defined as the saline infusion rate mu ltiplied by the intercontractile interval), void volume (VV), and non-voiding bladder contraction (NVC, intravesical pressure increases >5 mmHg during the filling phase, without the release of fluid from the urethra) and voiding efficiency (VE, voided volume/bladder capacity × 100) were determined (Vera and Nadelhaft, 2001; Füllhase et al., 2013; Lai et al., 2016; Wu et al., 2016). Estrous cycle phase in female mice was not recorded prior to conducting cystometry.

### Analysis of bladder innervation density

Sagittal cryo-sections were obtained of *Htr3a*^+/+^*, Htr3a*^+/−^, and *Htr3a*^−/−^ 18.5 dpc and adult male bladders were stained for markers of various neuronal subtypes: Tuj1 (all neurons), vesicular acetylcholine transporter (vAChT, cholinergic), CGRP (peptidergic nociceptive sensory), and NF200 (myelinated Aδ sensory). Three to five cryo-sections per animal, for each genotype class, were imaged. For each cryo-section, five images (665 × 508 μm) were captured: superior and inferior bladder neck (referred to as “Bladder Neck”), and superior, inferior, and apex bladder dome (referred to as “Bladder Dome”). All five images collectively are referred to as “Total Bladder.” Imaging was conducted on a Leica DMI6000B fluorescent microscope equipped with a 20x objective and software zoom set to 1.0x. Using ImageJ software (http://imagej.nih.gov/ij/) the grayscale images were processed using the threshold function to set the minimum gray value of the image that would be counted as true fluorescence and not background or auto-fluorescence. The same minimum gray value threshold was used for all images captured within one replicate of immunohistochemistry. To quantify innervation density in bladder smooth muscle, three non-contiguous 75 μm-diameter regions equivalent to 150 pixels were selected in each image and the ImageJ measure function was used to calculate the percentage of pixels in the selected area that were greater than or equal to the minimum gray value threshold. The percentages of pixels above the minimum gray value obtained from each randomly selected region were averaged for each image. Then, these values were averaged for each designated bladder domain (bladder dome, bladder neck, or total bladder).

### Statistical analysis

Average voiding frequency assessed by spontaneous void spot assay was averaged across 3 days of experiments and across animals segregated by sex and *Htr3a* genotype. The means were then statistically compared by one-way ANOVA followed by Tukey's Honest Significant Difference (HSD) *post-hoc* test to correct for multiple comparisons of pairs of means. A *p* < 0.05 was considered statistically significant. Anesthetized bladder cystometry data was averaged across animals segregated by sex and *Htr3a* genotype and then statistically compared by one-way ANOVA followed by Tukey's HSD *post-hoc* test. A *p* < 0.05 was considered statistically significant. Bladder smooth muscle innervation density was measured by calculating the percentage of pixels within a selected area that correspond to neuronal fiber staining. These measurements were averaged across male animals of each genotype (*Htr3a*^+/+^*, Htr3a*^+/−^, and *Htr3a*^−/−^), and means were statistically compared by one-way ANOVA followed by Tukey's HSD *post-hoc* test. A *p* < 0.05 was considered statistically significant. All numerical data are presented as the mean ± standard error of the mean (SEM). All study authors had access to study data, and all authors reviewed and approved the manuscript.

## Results

### Serotonin receptor 5-HT3A is expressed in mouse fetal and adult major pelvic ganglia

**Figure 1:**
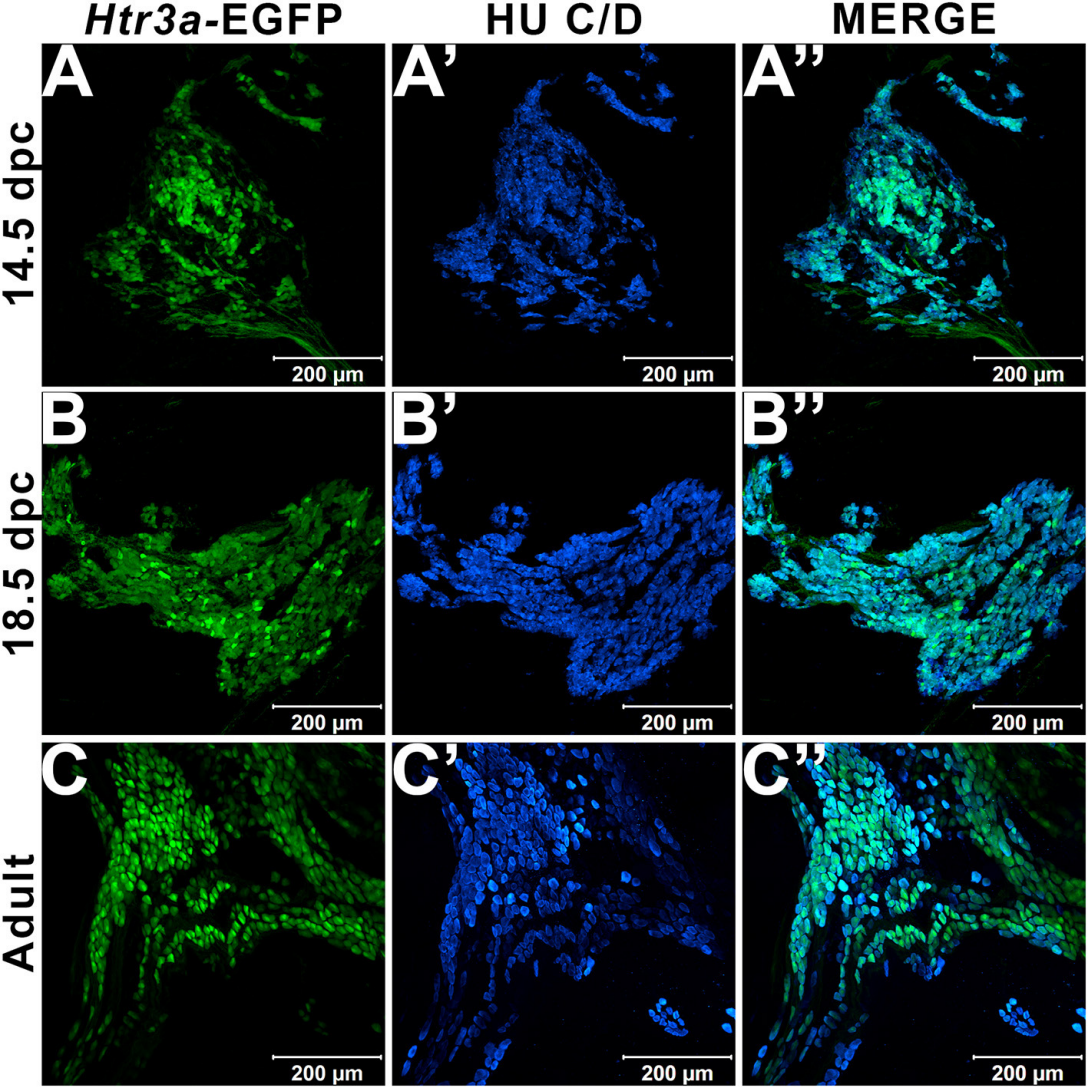
Serotonin receptor *Htr3a* is expressed in many neurons within fetal and adult major pelvic ganglia. Confocal images of *Htr3a-*EGFP pelvic ganglia stained with pan-neuronal marker Hu C/D. **(A**–**A”)** 14.5 dpc sagittal section of pelvic ganglia. **(B**–**B”)** 18.5 dpc sagittal section of pelvic ganglia. **(C**–**C”)** Adult (12 weeks old) male whole mount pelvic ganglia. Scale bar = 200 μm in all images.

In our previous study examining expression of *Htr3a* in developing dorsal root ganglia (Ritter and Southard-Smith, 2017), we also observed transgene fluorescence in the autonomic pelvic ganglia during fetal stages. To further characterize the expression of this serotonin receptor in developing autonomic LUT innervation, we evaluated the distribution and cell type specificity of *Htr3a* in *Htr3a-*EGFP transgenic tissues (Tg(*Htr3a-*EGFP)DH30Gsat/Mmnc, hereafter referred to as *Htr3a-*EGFP) by immunohistochemistry and confocal microscopy. We detected widespread *Htr3a-*EGFP co-expression with pan-neuronal marker Hu C/D in fetal pelvic ganglia at 14.5dpc (Figures 1A–A”). Expression of *Htr3a* is maintained through later fetal development in 18.5dpc pelvic ganglia neurons (Figures 1B–B”) and into adulthood (Figures 1C–C”). From these experiments we conclude that serotonin receptor 5-HT3A is expressed early in developing autonomic LUT innervation, and its expression is maintained in many pelvic ganglia neurons through sexual maturity in adult mice.

### *Htr3a^−/−^* males, but not females, exhibit increased urinary voiding frequency

**Figure 2:**
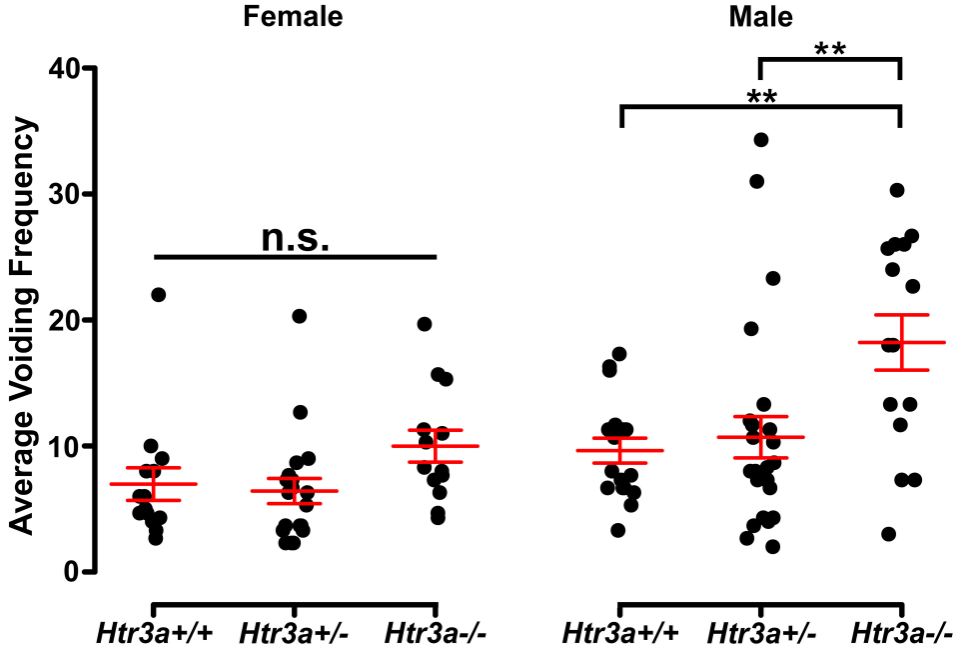
Male *Htr3a*^−/−^ mice, but not females, exhibit increased urinary voiding frequency in a spontaneous void spot assay. Plot of average voiding frequency (number of individual voids over the testing period) assessed by void spot assays in female and male *Htr3a*^+/+^*, Htr3a*^+/−^, and *Htr3a*^−/−^ 5-week old mice. Individual black dots represent different animals within each genotype class. The red lines indicate mean ± SEM. Female homozygous mutants trend toward increased voiding frequency but the difference in means is not statistically significant (*p* = 0.0911, one-way ANOVA). Male homozygous mutants, however, exhibit increased voiding frequency relative to wild type (*p* = 0.0046, one-way ANOVA with Tukey's HSD *post-hoc* test) and heterozygous (*p* = 0.0074, one-way ANOVA with Tukey's HSD *post-hoc* test) littermates. *n* = 13–25 animals per group. Asterisks denote the level of statistical significance: ^*^ < 0.05; ^**^ < 0.01; ^***^ < 0.001.

Given previous reports indicating a role for 5-HT3A receptor signaling in micturition (Bhattacharya et al., 2004; Schwen et al., 2013) and the widespread expression of *Htr3a* in pelvic ganglia neurons, we sought to determine if 5-HT3A is required for normal urinary voiding. To do this we employed the B6.129X1-*Htr3a*^*tm*1*Jul*^/J (hereafter *Htr3a*^−/−^) mouse line, which is a global knockout of *Htr3a* gene expression that has been bred to congenicity on the C57BL/6J inbred strain (Zeitz et al., 2002). Bladder function was initially assessed in *Htr3a* mutants and wild type littermates by spontaneous void spot assays (VSA) that have been shown in prior studies to be a reliable assessment of micturition in mice (Yu et al., 2014; Bjorling et al., 2015; Keil et al., 2016). In 1 h assessments conducted across 3 consecutive days, we observed male wild type and heterozygous animals had very similar voiding frequencies (9.63 ± 0.98 voids and 10.69 ± 1.64 voids, respectively, *p* = 0.8894, one-way ANOVA with Tukey's HSD *post-hoc* test), while homozygous mutant males exhibited an increased number of voids that averaged 18.21 ± 2.19 (Figure 2). The difference in *Htr3a*^−/−^ male voiding frequencies was significantly increased compared to both wild type and *Htr3a*^+/−^ littermates (*p* = 0.0046 and *p* = 0.0074, respectively, one-way ANOVA with Tukey's HSD *post-hoc* test, *n* = 15–25 animals per genotype). However, wild type, heterozygous, and *Htr3a* female homozygous mutants did not differ significantly in their average voiding frequencies despite a trending increase in void frequency in female *Htr3a*^−/−^ mice that trends in the same direction as that seen for *Htr3a*^−/−^ males (6.97 ± 1.29, 6.42 ± 0.99, and 9.98 ± 1.27 voids, respectively, *p* = 0.09108, one-way ANOVA, *n* = 13–19 animals per genotype). Total void surface area averaged over three trial days was not significantly different among *Htr3a* genotypes in males or females (data not shown). From these experiments we conclude that male *Htr3a*^−/−^ mutant mice have more frequent urinary voids.

### Male *Htr3a^−/−^* urinary voiding efficiency is impaired

**Figure 3:**
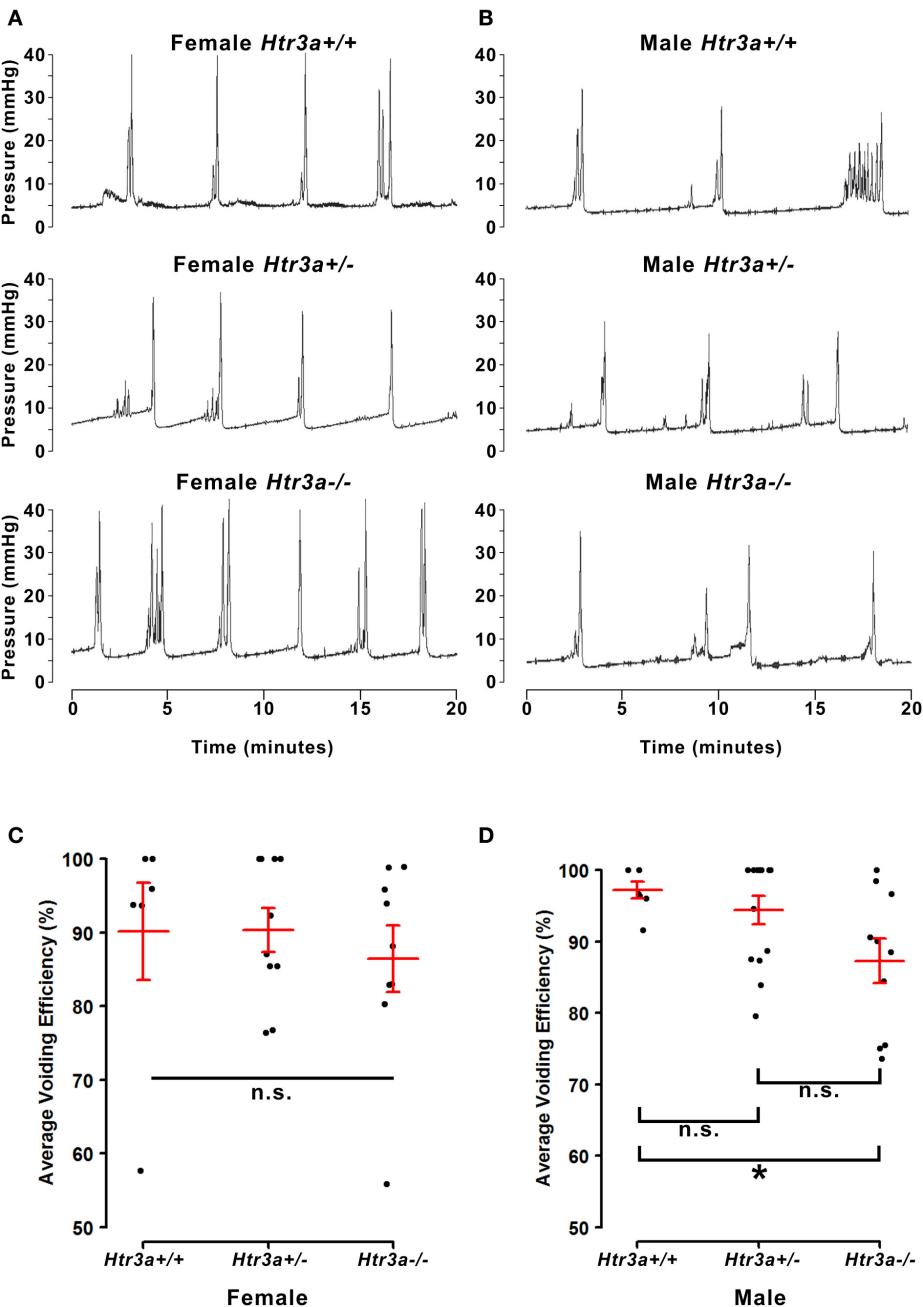
Male, but not female *Htr3a*^−/−^ mice demonstrate decreased bladder voiding efficiency compared to wild type mice. Voiding efficiency, calculated as the percentage bladder capacity expelled per void, was measured in adult *Htr3a*KO animals *in vivo* via anesthetized bladder cystometry. Intraluminal bladder pressure (mmHg) was measured over time (minutes) *in vivo* in **(A)** female adult mice, and **(B)** male adult mice. **(C)** Jitter plot of voiding efficiency in female *Htr3a*^+/+^*, Htr3a*^+/−^, and *Htr3a*^−/−^ adult mice. Red lines indicate mean ± SEM. The difference in voiding efficiency in female mice was not significant (*p* = 0.7701, one-way ANOVA). **(D)** Jitter plot of voiding efficiency in male *Htr3a*^+/+^*, Htr3a*^+/−^, and *Htr3a*^−/−^ adult mice. *Htr3a*^−/−^ males demonstrate a significant decrease in voiding efficiency compared to wild type littermates (*p* = 0.0357, one-way ANOVA with Tukey's HSD *post-hoc* test). The difference between heterozygotes and wildtype littermates was not significant (*p* = 0.7068, one-way ANOVA with Tukey's HSD *post-hoc* test), nor was the difference between heterozygotes and homozygotes (*p* = 0.0826, one-way ANOVA with Tukey's HSD *post-hoc* test). *n* = 6–14 per group.

The 5-HT3A receptor is expressed not only in the autonomic pelvic ganglia and sensory dorsal root ganglia supplying bladder innervation, but also in other aspects of the micturition circuitry. Recent work has shown that 5-HT3A is expressed in Barrington's nucleus, the locus coeruleus, periaqueductal gray, and entorhinal cortex (Koyama et al., 2017), all of which have been previously implicated in relaying autonomic output and cortical regulation of voiding behavior (Fowler et al., 2008). Because the *Htr3a*^−/−^ mutant line used in our studies is a global knockout, micturition centers in the brain expressing 5-HT3A may also be affected. To determine if the effects of 5-HT3A loss observed in the spontaneous void spot assay are due to autonomic dysfunction or cognitive control of voiding behavior, we conducted anesthetized bladder cystometry *in vivo* in female and male *Htr3a*^+/+^*, Htr3a*^+/−^, and *Htr3a*^−/−^ adult mice. Anesthesia of the animals during cystometry allows isolation of involuntary urinary voiding and eliminates the behavioral component of micturition. Parameters measured during anesthetized cystometry included bladder volume-to-body weight ratio, intercontractile interval, maximal voiding pressure, void volume, bladder capacity, and non-voiding bladder contractions. There were no significant differences between any of the *Htr3a* genotype classes for these measures among either male or female mice (Table 3). Representative cystometric traces that illustrate comparable values are shown in Figures 3A,B. In addition, we measured voiding efficiency, which is defined as the percentage of bladder capacity expelled in one voiding event (Figure 3C). Normally, an efficient bladder will expel its entire bladder capacity in one voiding event, which would be reflected in a voiding efficiency value of nearly 100%. Wild type male mice exhibited an average voiding efficiency of 97.24 ± 3.01% while heterozygous mice had a voiding efficiency of 94.39 ± 1.98% and homozygous mutant voiding efficiency was 87.30 ± 3.12% (*n* = 7–14 animals per genotype). This level of voiding efficiency was significantly different for male *Htr3a*^−/−^ mice compared to wild type littermates (*p* = 0.0357, one-way ANOVA with Tukey's HSD *post-hoc* test). Despite the significant difference in voiding efficiency for homozygous mice, voiding efficiency of heterozygous mice was not significantly different from wild type or homozygous littermates (*p* = 0.7068 and *p* = 0.0826 respectively, one-way ANOVA with Tukey's HSD *post-hoc* test). Average voiding efficiencies of *Htr3a*^+/+^*, Htr3a*^+/−^, and *Htr3a*^−/−^ females were similar, with values of 90.19 ± 6.60, 90.35 ± 3.01, and 86.43 ± 4.49%, respectively (*p* = 0.7701, one-way ANOVA, *n* = 6–10 animals per genotype). Given the detrimental effect of *Htr3a* gene loss on the ability of homozygous males to efficiently empty bladder content, we conclude that 5-HT3A is required for maintaining normal LUT function in male mice.

**Table 3:** Summary of anesthetized bladder cystometry measurements.

**Cystometric parameter**	**Female *Htr3a^+/+^***	**Female *Htr3a^+/−^***	**Female *Htr3a*^−/−^**	**One-way ANOVA p-value**	**Male *Htr3a^+/+^***	**Male *Htr3a^+/−^***	**Male *Htr3a*^−/−^**	**One-way ANOVA *p*-value**
Voiding Efficiency	90.19%±6.60%	90.35 ± 3.01%	86.43 ± 4.49%	0.7701	97.24%±3.01%	94.39 ± 1.98%	87.30%±3.12%	0.0287^*^
Bladder Volume/Body Weight Ratio (mm^3^/g)	33.96 ± 6.92	32.25 ± 5.71	34.88 ± 7.99	0.9618	78.48 ± 22.23	64.51 ± 10.89	38.97 ± 7.90	0.2000
Bladder Capacity (μL)	54.43 ± 8.01	63.69 ± 6.55	80.97 ± 17.33	0.3427	77.55 ± 11.55	69.81 ± 11.18	75.51 ± 16.66	0.9153
Intercontractile Interval (sec)	244.92 ± 36.06	286.59 ± 29.49	364.44 ± 77.99	0.3423	348.41 ± 51.98	314.17 ± 50.31	339.80 ± 74.96	0.9154
Void Volume (μL)	50.38 ± 8.99	58.32 ± 6.75	67.17 ± 12.94	0.5490	77.84 ± 11.61	66.58 ± 10.21	64.60 ± 13.61	0.7609
Maximum Void Pressure (mmHg)	26.62 ± 3.75	28.56 ± 1.09	32.09 ± 1.26	0.1556	20.53 ± 1.38	21.40 ± 1.36	23.38 ± 2.20	0.5388
Non-Voiding Contractions	0.083 ± 0.083	0.280 ± 0.207	0.478 ± 0.329	0.5979	1.39 ± 0.41	1.40 ± 0.38	1.01 ± 0.33	0.7178

*Anesthetized bladder cystometry was conducted to determine the effects of 5-HT3A receptor loss on unconscious voiding activity. Mean values ± SEM are given for female and male wild type, heterozygous, and homozygous Htr3a knockout adult mice. One-way ANOVA was conducted to determine if differences in means were significant among female mice and male mice. The statistically significant p-value (<0.05) is presented in bold with an asterisk. Voiding efficiency is defined as the percent bladder capacity expelled in a voiding event. Bladder volume-to-body weight ratio was calculated by dividing the bladder volume (mm^3^) by the animal's total body weight. Bladder capacity is defined as the intervoid interval multiplied by the infusion rate of saline during the assay. Intercontractile interval is defined as the time (in seconds) between voiding events. Void volume is defined as the volume of infusate voided during a micturition event. Maximum void pressure is defined as the peak intravesical bladder pressure immediately prior to micturition. Non-voiding contractions are defined as rhythmic increases in intravesical pressure that occur between micturition events*.

### Loss of 5-HT3A disrupts autonomic neuronal marker expression patterns in fetal pelvic ganglia

**Figure 4:**
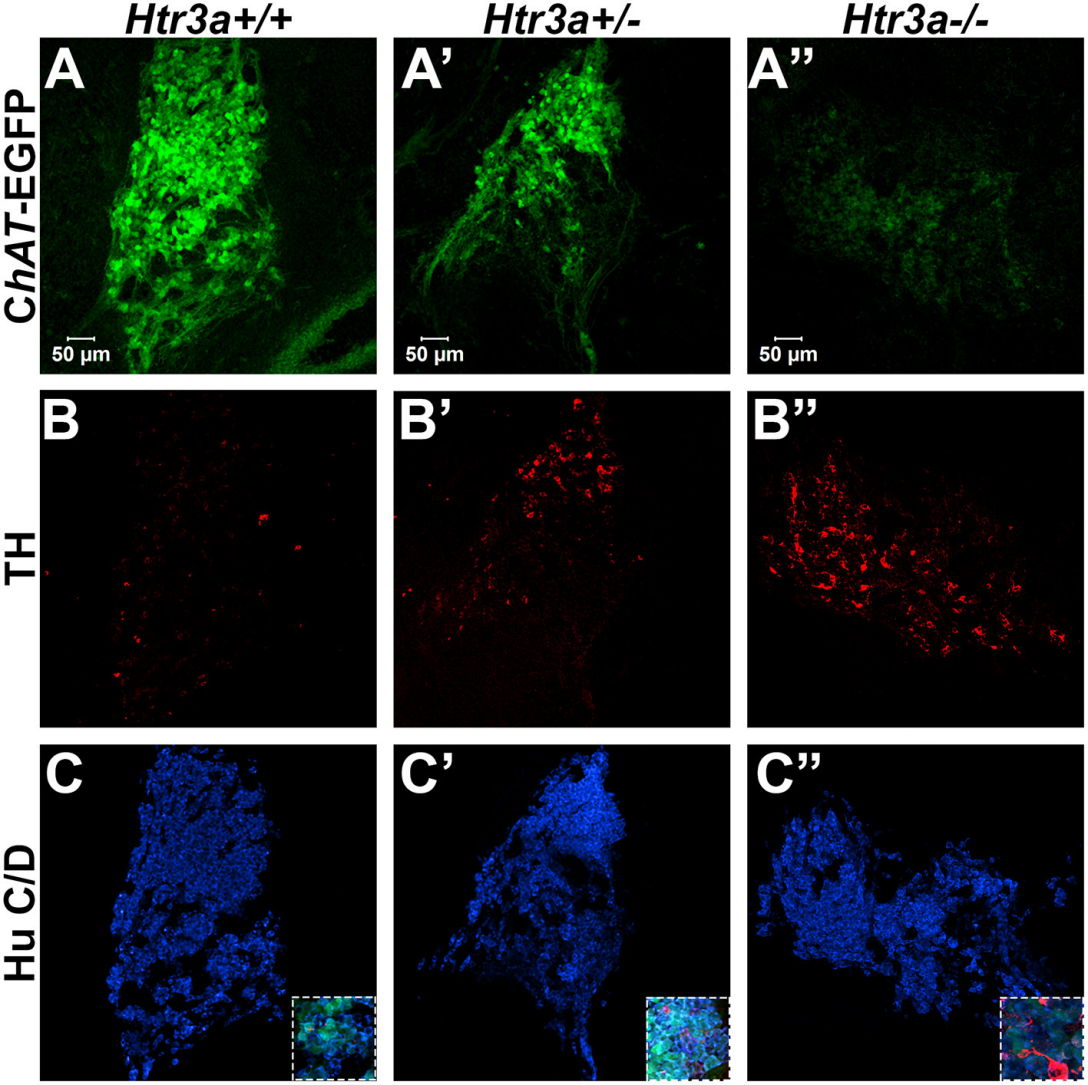
Loss of serotonin receptor *Htr3a* increases expression of tyrosine hydroxylase in fetal pelvic ganglia. Confocal images of sagittal cryo-sections of male 14.5 dpc *ChAT-*EGFP; *Htr3a*KO pelvic ganglia stained with adrenergic neuronal marker tyrosine hydroxylase (TH) and pan-neuronal marker Hu C/D. **(A**–**A”)** *ChAT-*EGFP, labeling cholinergic neurons, is markedly reduced in *Htr3a* mutant fetal pelvic ganglia compared to wild type. **(B**–**B”)** TH, labeling adrenergic neurons, is increased in *Htr3a* mutant fetal pelvic ganglia compared to wild type. **(C**–**C”)** Expression of pan-neuronal marker Hu C/D is similar among all three genotypes examined at 14.5 dpc. Insets show 5X zoom of merged images of all three markers. *n* = 4 animals per genotype.

**Figure 5:**
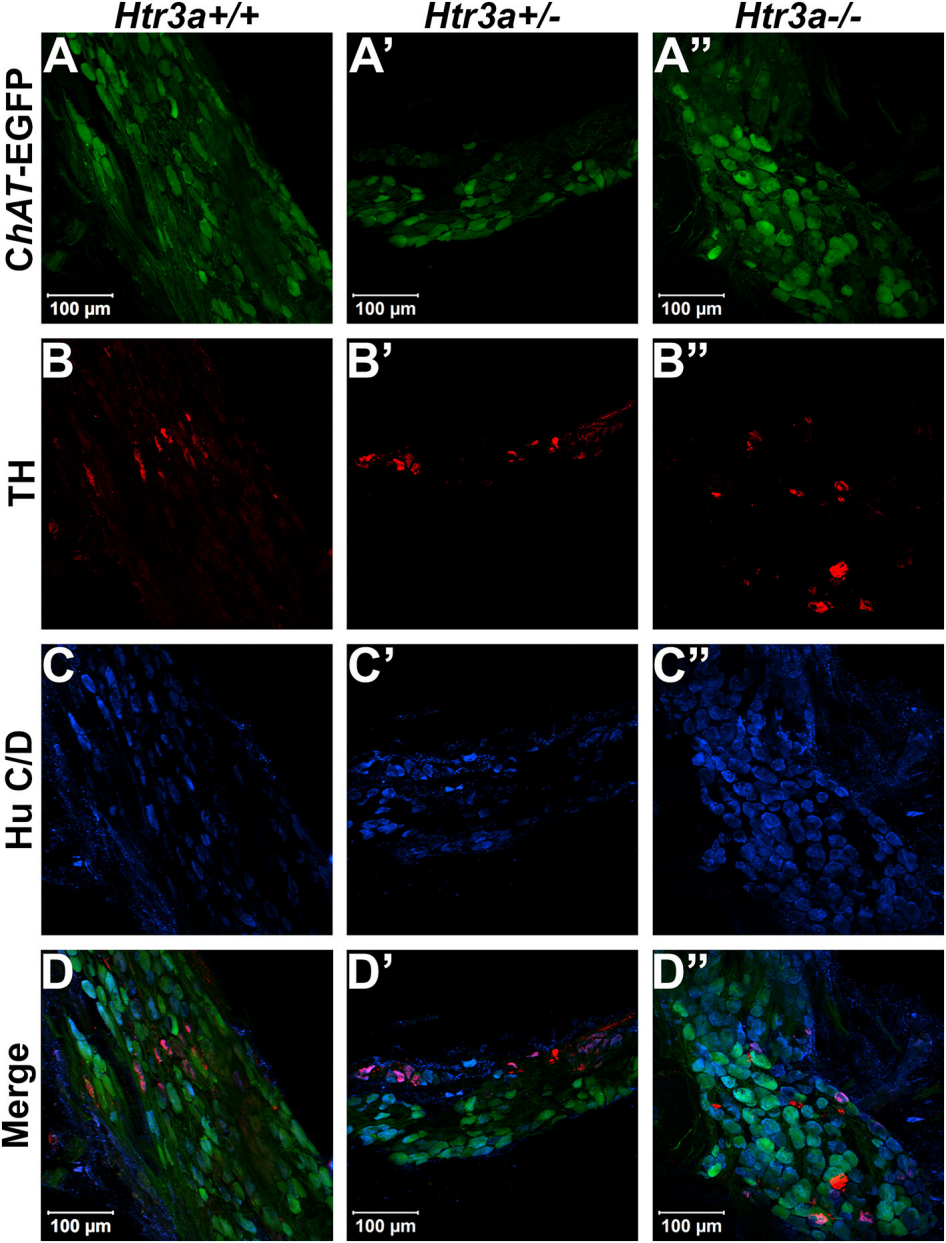
Autonomic marker expression patterns are normal in adult *Htr3a* mutant pelvic ganglia. Confocal images of cryo-sections of adult (6 weeks old) male *ChAT-*EGFP; *Htr3a*KO pelvic ganglia stained with adrenergic neuronal marker TH and pan-neuronal marker Hu C/D. Expression patterns of **(A**–**A”)** *ChAT*-EGFP; **(B**–**B”)** TH; and **(C**–**C”)** Hu C/D are similar among *Htr3a*^+/+^*, Htr3a*^+/−^, and *Htr3a*^−/−^ pelvic ganglia. **(D**–**D”)** merged images of panels **(A–C**, **A'–C', A”–C”)**. *n* = 6 animals per genotype.

Given the consequences of *Htr3a* gene loss on adult bladder function and our observation of *Htr3a* expression in fetal mouse pelvic ganglia, we examined the effects of *Htr3a* loss on the development of pelvic ganglia neurons. To assess the composition of pelvic ganglia during development, we monitored the appearance of cholinergic neurons that comprise the majority of pelvic ganglia neuronal subtypes (Wanigasekara et al., 2003) as well as noradrenergic neurons labeled by tyrosine hydroxylase (TH) expression. *Htr3a* mutants were crossed with the Tg(*ChAT-*EGFP)GH293Gsat/Mmucd (hereafter *ChAT*-EGFP) transgenic reporter mouse line (Gong et al., 2003) to facilitate imaging of cholinergic neurons, as direct visualization by GFP labeled discrete cholinergic neurons more readily than several commercially available ChAT antisera (data not shown). Progeny from intercrosses of *ChAT-*EGFP; *Htr3a*^+/−^ mice were sectioned sagittally and stained for TH and the pan-neuronal marker Hu C/D. At 14.5 dpc, we observed a visible reduction in *ChAT*-EGFP expression in *Htr3a*^+/−^ and *Htr3a*^−/−^ pelvic ganglia compared to *Htr3a*^+/+^ littermates, along with a concurrent increase in TH expression (*n* = 4 animals per genotype, Figure 4). However, when we examined expression of these markers in adult (6–8 weeks old) male and female pelvic ganglia, the differences between *Htr3a* genotypes seen at 14.5 dpc were no longer apparent among multiple replicates examined (*n* = 6 animals per genotype, Figure 5). Expression of TH in adult pelvic ganglia across all three genotype classes was comparable to prior reports of noradrenergic neurons in adult mice (Yan and Keast, 2008). From these experiments we conclude that loss of 5-HT3A receptor produces a transient disruption of expression of autonomic neuronal subtype markers in fetal pelvic ganglia.

### *Htr3a* mutant bladder detrusor is more densely innervated compared to wild type

The 5-HT3A receptor has been previously implicated in mediating neuronal morphological complexity in the developing mouse brain (Chameau et al., 2009; Oostland et al., 2013). Specifically, *Htr3a*^−/−^ mice exhibit hyper-elaboration of pyramidal neuronal dendritic fibers in the developing cortex; however, its role in peripheral neuronal development *in vivo* has not been reported. To determine if loss of 5-HT3A alters aspects of peripheral neuronal development or neuronal morphology, we conducted immunohistochemistry and measured innervation density of bladder smooth muscle on sagittal sections from 18.5dpc *Htr3a*^+/+^*, Htr3a*^+/−^, and *Htr3a*^−/−^ mice (*n* = 5 animals per genotype). After staining for a variety of neuronal markers, we measured the percentage of pixels of a minimum intensity level within a fixed area of bladder smooth muscle for three domains (total bladder, bladder neck, and bladder dome) as a proxy for average area innervated by labeled neuronal fibers. These measurements, along with statistical *p*-values, are listed in Table 4.

**Table 4:** Measurements of 18.5dpc bladder smooth muscle innervation density.

**Neuronal Marker**		**Percentage Innervation Density in 18.5 dpc Fetal Mice (average** ± **SEM)**			**One-way ANOVA**	**One-way ANOVA with Tukey's** ***post-hoc*** **HSD** ***p*****-values**		
		***Htr3a^+/+^***	***Htr3a^+/−^***	***Htr3a^−/−^***	**Overall**	**+/+ vs. +/−**	**+/− vs. −/−**	**+/+ vs. −/−**
Tuj1	Total	9.87 ± 1.15	16.22 ± 1.57	17.82 ± 1.58	**0.0052**	**0.0232**	0.7193	**0.0057**
	Neck	10.67 ± 1.76	18.27 ± 1.52	18.99 ± 2.17	**0.0135**	**0.0315**	0.9595	0.1928
	Dome	9.34 ± 0.82	14.76 ± 1.80	17.04 ± 1.38	**0.0061**	**0.0432**	0.4997	**0.0055**
vAChT	Total	7.23 ± 1.71	15.06 ± 4.19	18.47 ± 2.33	**0.0316**	0.1601	0.6706	**0.0283**
	Neck	7.80 ± 1.94	13.95 ± 3.07	19.28 ± 3.14	**0.0335**	0.3081	0.4029	**0.0269**
	Dome	6.85 ± 1.63	14.67 ± 4.10	17.92 ± 1.99	**0.0248**	0.1327	0.6637	**0.0223**
CGRP	Total	1.29 ± 0.24	1.43 ± 0.34	2.38 ± 0.78	0.2986	N/A	N/A	N/A
	Neck	1.62 ± 0.35	1.94 ± 0.46	3.18 ± 1.05	0.2777	N/A	N/A	N/A
	Dome	1.08 ± 0.18	1.08 ± 0.28	1.84 ± 0.63	0.3612	N/A	N/A	N/A
NF200	Total	8.05 ± 1.69	10.07 ± 1.92	11.42 ± 2.32	0.5118	N/A	N/A	N/A
	Neck	9.13 ± 1.85	10.29 ± 1.77	12.92 ± 2.35	0.4284	N/A	N/A	N/A
	Dome	7.33 ± 1.78	9.92 ± 2.08	10.42 ± 2.31	0.5488	N/A	N/A	N/A

*Innervation density was measured by calculating the percentage of pixels in a selected region of 18.5 dpc bladder smooth muscle that exceeded the threshold intensity level for neuronal fiber staining. Average percentages were calculated from values obtained from images of superior and inferior bladder neck (“Bladder Neck”), and superior, inferior, and apex of bladder dome (“Bladder Dome”). “Total Bladder” innervated density was calculated by averaging the percentage values from both bladder neck and bladder dome. Overall differences in means were statistically compared with one-way ANOVA; Tukey's Honest Significant Difference (HSD) post-hoc test was conducted to compare pairs of means. Statistically significant p-values (p < 0.05) are presented in bold*.

**Figure 6:**
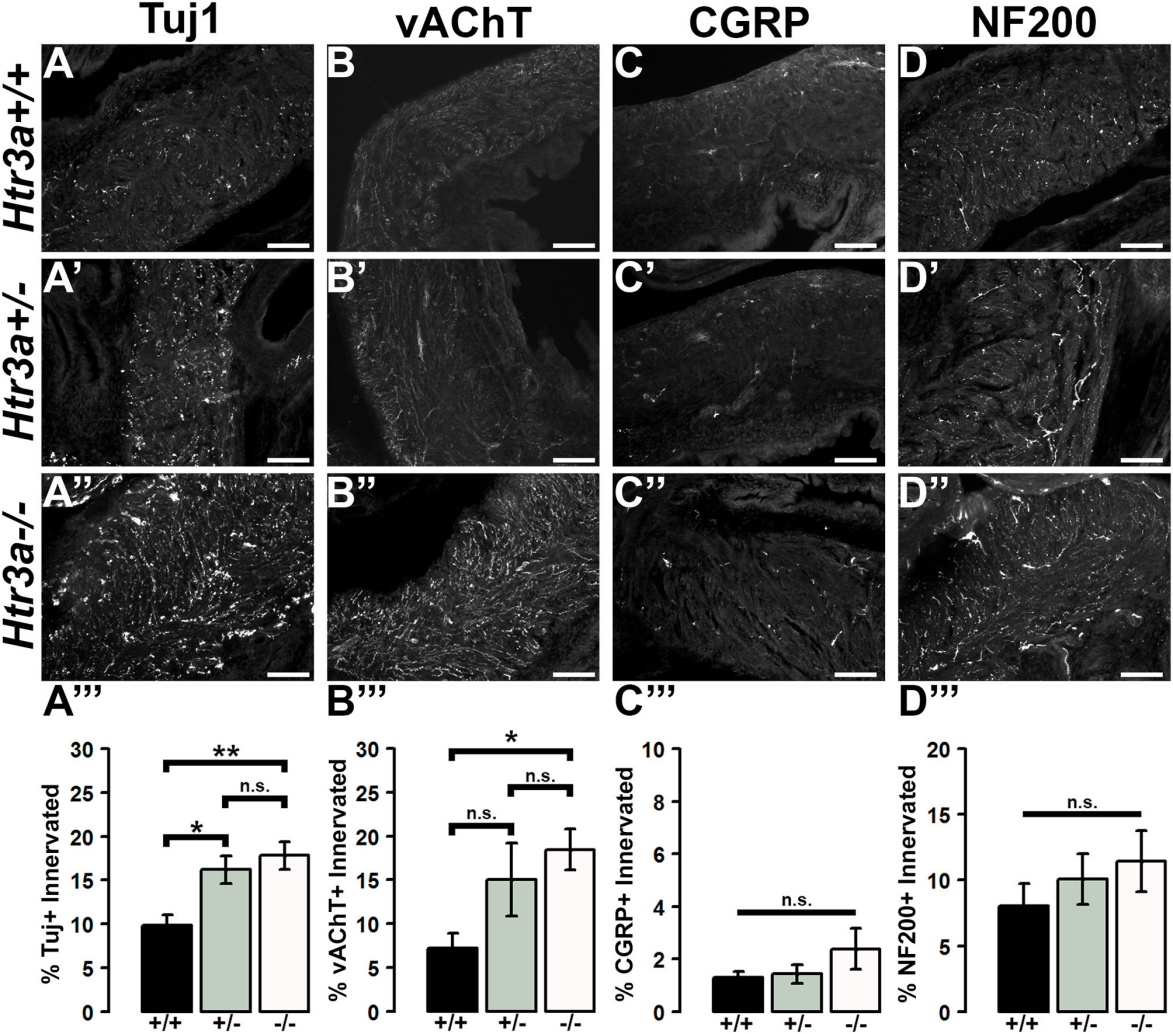
Loss of serotonin receptor *Htr3a* leads to increased density of autonomic innervation in late fetal bladder smooth muscle. Sagittal cryo-sections of *Htr3a*^+/+^*, Htr3a*^+/−^, and *Htr3a*^−/−^ 18.5 dpc bladders stained with neuronal markers. **(A**–**A”)** Tuj1, pan-neuronal marker; **(B**–**B”)** vesicular acetylcholine transporter (vAChT), marker of cholinergic autonomic neurons; **(C**–**C”)** Calcitonin Gene Related Peptide (CGRP), marker of peptidergic sensory neurons; **(D**–**D”)** Neurofilament 200 (NF200), marker of Aδ mechanosensory neurons. Innervation density was quantified by measuring the percentage of pixels within fixed areas of bladder smooth muscle images that met the gray value threshold indicative of neuronal fiber staining. Bar plots of innervation density illustrate quantification of combined superior and inferior bladder neck and superior, inferior, and apex of bladder dome innervated ± SEM by Tuj1+ **(A”')**, vAChT+ **(B”')**, CGRP+ **(C”')**, and NF200+ **(D”')** fibers. Overall differences in means were assessed by one-way ANOVA; statistical significance of differences between pairs of means are listed in Table 3. *p* < 0.05 was considered significant. *n* = 5 animals per genotype. Scale bar = 100 μm. Asterisks denote the level of statistical significance: ^*^ < 0.05; ^**^ < 0.01; ^***^ < 0.001.

We first asked if overall innervation patterns differed between wild type, heterozygous, and *Htr3a*^−/−^ fetal tissues by staining for the pan-neuronal marker Tuj1 (β-III tubulin) that strongly labels neuronal fibers. Interestingly, Tuj1+ staining was significantly increased in all areas of bladder smooth muscle examined in *Htr3a*^+/−^ and *Htr3a*^−/−^ samples compared to wild type littermates (*p* = 0.0232 and *p* = 0.0057 respectively, one-way ANOVA with Tukey's HSD *post-hoc* test). The average innervation density in wild type bladders was 9.87 ± 1.15%, while *Htr3a*^+/−^ and *Htr3a*^−/−^ Tuj1+ innervation densities were nearly doubled (16.22 ± 1.57 and 17.82 ± 1.58%, respectively) (Figures 6A–A”). The difference between *Htr3a*^+/−^ and *Htr3a*^−/−^ Tuj1+ innervation density measurements was not statistically significant (*p* = 0.7193, one-way ANOVA with Tukey's HSD *post-hoc* test).

Given the large proportion of cholinergic neurons found in the autonomic pelvic ganglia and their critical role in mediating bladder contractility (de Groat and Yoshimura, 2015), we next asked if 18.5 dpc *Htr3a* mutants would exhibit perturbed cholinergic innervation patterns in the bladder smooth muscle. Upon staining with vesicular acetylcholine transporter (vAChT), we observed that 7.23 ± 1.71% of the wildtype bladder smooth muscle received vAChT+ innervation, while average innervation density in *Htr3a*^+/−^ and *Htr3a*^−/−^ animals was 15.06 ± 4.19 and 18.47 ± 2.33% respectively. The increase in *Htr3a*^−/−^ vAChT+ bladder innervation density was significant compared to wild type bladder (*p* = 0.0283, one-way ANOVA with Tukey's HSD *post-hoc* test), but *Htr3a*^+/−^ innervation density was not significantly different compared to either wild type or homozygous bladder (*p* = 0.1601 and *p* = 0.6706 respectively, one-way ANOVA with Tukey's HSD *post-hoc* test) (Figures 6B–B”).

The sensation of bladder filling and muscle stretch is primarily detected by sensory afferent neurons in the dorsal root ganglia (de Groat and Yoshimura, 2015). During our prior analysis of *Htr3a* expression in the developing dorsal root ganglia, we documented that many bladder-innervating sensory neurons expressing *Htr3a* also express neuropeptide Calcitonin Gene Related Peptide (CGRP) and mechanosensory marker Neurofilament 200 (NF200) (Ritter and Southard-Smith, 2017). We were interested to see if these populations would be affected by loss of *Htr3a*. Upon staining for CGRP, we did not observe any significant differences in CGRP+ innervation density of bladder at 18.5 dpc (*p* = 0.2986, one-way ANOVA) (Figures 6C–C”). CGRP+ innervation density in *Htr3a*^+/+^*, Htr3a*^+/−^, and *Htr3a*^−/−^ averaged 1.29 ± 0.24, 1.43 ± 0.34, and 2.38 ± 0.78%, respectively. Similarly, fetal bladders across these three genotypes did not differ significantly in NF200+ innervation density (Figures 6D–D”). Innervation density of NF200+ fibers were 8.05 ± 1.69, 10.01 ± 1.92, and 11.42 ± 2.32% for *Htr3a*^+/+^*, Htr3a*^+/−^, and *Htr3a*^−/−^, respectively (*p* = 0.5118, one-way ANOVA). From these experiments we conclude that in late fetal development, loss of *Htr3a* gene expression results in an overall increase in the presence of neuronal fibers in fetal bladder smooth muscle, with cholinergic fibers being particularly enriched while sensory fibers are not at this stage of development.

**Figure 7:**
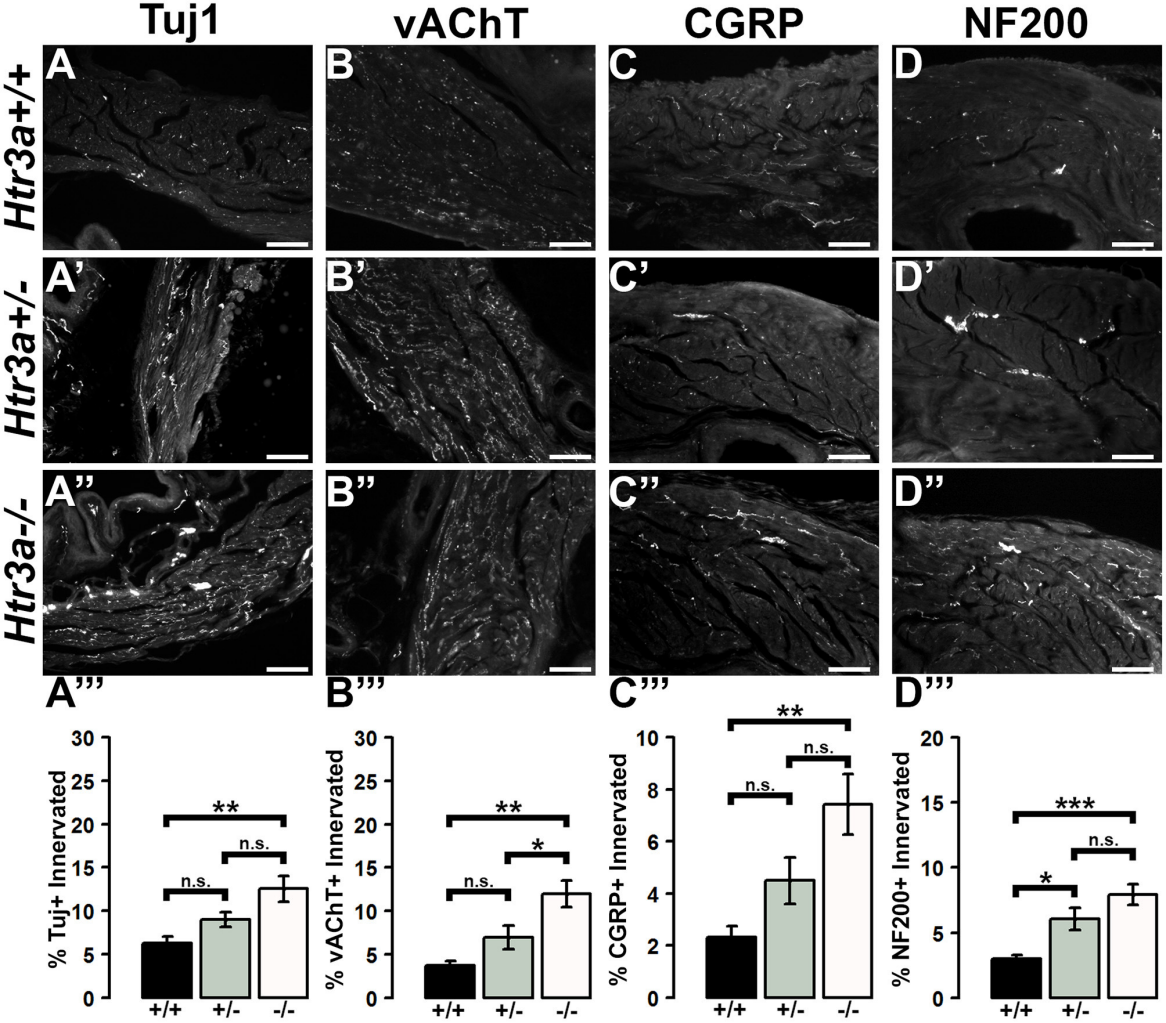
Loss of serotonin receptor *Htr3a* leads to increased density of autonomic and sensory innervation in adult bladder smooth muscle. Sagittal cryo-sections of *Htr3a*^+/+^*, Htr3a*^+/−^, and *Htr3a*^−/−^ adult male bladders stained with neuronal markers. **(A**–**A”)** Tuj1, pan-neuronal marker; **(B**–**B”)** vesicular acetylcholine transporter (vACHT), marker of autonomic cholinergic neurons; **(C**–**C”)** Calcitonin Gene Related Peptide (CGRP), marker of peptidergic sensory neurons; **(D**–**D”)** Neurofilament 200 (NF200), marker of Aδ mechanosensory neurons. Innervation density was quantified by measuring the percentage of pixels within fixed areas of bladder smooth muscle images that met the gray value threshold indicative of neuronal fiber staining. Bar plots of innervation density illustrate quantification of combined superior and inferior bladder neck and superior, inferior, and apex of bladder dome innervated ± SEM by Tuj1+ **(A”')**, vAChT+ **(B”')**, CGRP+ **(C”')**, and NF200+ **(D”')** fibers. Overall differences in means were assessed by one-way ANOVA; statistical significance of differences between pairs of means are listed in Table 3. *p* < 0.05 was considered significant. *n* = 5 animals per genotype. Scale bar = 100 μm. Asterisks denote the level of statistical significance: ^*^ < 0.05; ^**^ < 0.01; ^***^ < 0.001.

Due to the refining of neural architecture that may occur over the postnatal developmental period (Purves and Lichtman, 1980; Tapia et al., 2012), we sought to determine if differences in innervation patterns would also be present in adult *Htr3a* mutants. We conducted the same staining and analysis procedures on adult male *Htr3a*^+/+^*, Htr3a*^+/−^, and *Htr3a*^−/−^ bladder sagittal sections (*n* = 5 animals per genotype, Table 5). Quantification of Tuj1+ staining in adult bladder tissues similarly revealed a significant increase in innervation density in *Htr3a*^−/−^ mutant bladder compared to wild type (*p* = 0.0033, one-way ANOVA with Tukey's HSD *post-hoc* test) (Figures 7A–A”). The average adult wild type Tuj1+ innervation density was 6.32 ± 0.72%, while *Htr3a*^+/−^ bladders were 9.01 ± 0.81% innervated and *Htr3a*^−/−^ bladders exhibited a two-fold increase in innervation density, with average percent innervated values of 12.54 ± 1.49%. The difference between *Htr3a*^+/−^ and wild type Tuj1 innervation density was not significant (*p* = 0.2082, one-way ANOVA with Tukey's HSD *post-hoc* test), nor was the difference between *Htr3a*^+/−^ and *Htr3a*^−/−^ Tuj1 innervation density (*p* = 0.0828, one-way ANOVA with Tukey's HSD *post-hoc* test).

**Table 5:** Measurements of adult bladder smooth muscle innervation density.

**Neuronal Marker**		**Percentage Innervation Density in Adult Mice (average** ± **SEM)**			**One-way ANOVA**	**One-way ANOVA with Tukey's** ***post-hoc*** **HSD** ***p*****-values**		
		***Htr3a^+/+^***	***Htr3a^+/−^***	***Htr3a^−/−^***	**Overall**	**+/+ vs. +/−**	**+/− vs. −/−**	**+/+ vs. −/−**
Tuj1	Total	6.32 ± 0.72	9.01 ± 0.81	12.54 ± 1.49	**0.0044**	0.2082	0.0828	**0.0033**
	Neck	6.89 ± 0.57	9.89 ± 1.09	12.38 ± 0.94	**0.0034**	0.0832	0.1631	**0.0026**
	Dome	5.94 ± 0.87	8.51 ± 1.01	12.64 ± 2.42	**0.0351**	0.5106	0.2016	**0.0294**
vAChT	Total	3.74 ± 0.54	6.94 ± 1.36	11.98 ± 1.57	**0.0018**	0.2041	**0.0344**	**0.0014**
	Neck	4.19 ± 0.95	7.56 ± 1.84	12.04 ± 1.74	**0.0131**	0.3130	0.1477	**0.0102**
	Dome	3.43 ± 0.39	6.59 ± 1.15	11.95 ± 1.62	**0.0008**	0.1789	**0.0183**	**0.0006**
CGRP	Total	2.32 ± 0.43	4.49 ± 0.89	7.43 ± 1.17	**0.0052**	0.2323	0.0867	**0.0039**
	Neck	2.53 ± 0.43	5.07 ± 0.85	8.71 ± 1.97	**0.0153**	0.3605	0.1459	**0.0122**
	Dome	2.18 ± 0.49	4.10 ± 1.03	6.59 ± 0.77	**0.0069**	0.2426	0.1082	**0.0053**
NF200	Total	3.01 ± 0.29	6.06 ± 0.83	7.92 ± 0.79	**0.0009**	**0.0219**	0.1759	**0.0007**
	Neck	4.26 ± 0.68	5.45 ± 0.80	9.59 ± 1.33	**0.0057**	0.6754	**0.0279**	**0.0061**
	Dome	2.18 ± 0.12	6.42 ± 1.08	6.81 ± 0.79	**0.0019**	**0.0059**	0.9316	**0.0031**

*Innervation density was measured by calculating the percentage of pixels in a selected region of adult bladder smooth muscle that exceeded the threshold intensity level for neuronal fiber staining. Average percentages were calculated from values obtained from images of superior and inferior bladder neck (“Bladder Neck”), and superior, inferior, and apex of bladder dome (“Bladder Dome”). “Total Bladder” innervated density was calculated by averaging the percentage values from both bladder neck and bladder dome. Overall differences in means were statistically compared with one-way ANOVA; Tukey's Honest Significant Difference (HSD) post-hoc test was conducted to compare pairs of means. Statistically significant p-values (p < 0.05) are presented in bold*.

When we stained for vAChT to visualize cholinergic fibers in adult bladder tissues we noted that *Htr3a* mutants maintained higher than normal levels of cholinergic fiber density into adulthood, and the difference among genotypes was more pronounced than in fetal samples. Adult *Htr3a*^+/+^ bladder smooth muscle tissues had an average vAChT+ innervation density of 3.74 ± 0.54% (Figures 7B–B”). In contrast, *Htr3a*^+/−^ and *Htr3a*^−/−^ bladder tissues had an average innervation density of 6.94 ± 1.36 and 11.98 ± 1.57%, respectively. *Htr3a*^−/−^ vAChT+ innervation density was significantly increased compared to both wild type and heterozygous littermates (*p* = 0.0014 and *p* = 0.0344 respectively, one-way ANOVA with Tukey's HSD *post-hoc* test), but *Htr3a*^+/−^ vAChT+ innervation was not significantly different from wild type (*p* = 0.2041, one-way ANOVA with Tukey's HSD *post-hoc* test).

Quantification of sensory innervation density by immunohistochemical localization of CGRP+ fibers revealed that, in contrast to density of sensory innervation in fetal tissues, bladder smooth muscle of adult *Htr3a*^−/−^ mutants is more densely innervated by sensory fibers than that of wild type (*p* = 0.0039, one-way ANOVA with Tukey's HSD *post-hoc* test) (Figures 7C–C”). Wild type bladder had an average CGRP+ innervation density of 2.32 ± 0.43%, while *Htr3a*^+/−^ innervation density levels were 4.49 ± 0.89% and *Htr3a*^−/−^ CGRP+ innervation was increased approximately three-fold, values of 7.43 ± 1.17%. *Htr3a*^+/−^ CGRP+ innervation density was not significantly different from either wild type or *Htr3a*^−/−^ tissues (*p* = 0.2323 and *p* = 0.0867 respectively, one-way ANOVA with Tukey's HSD *post-hoc* test).

Staining adult bladder tissues with NF200 also revealed a highly significant increase in mechanosensory neuronal innervation of both adult *Htr3a*^+/−^ and *Htr3a*^−/−^ mutant bladder smooth muscle compared to wild type (*p* = 0.0219 and *p* = 0.0007 respectively, one-way ANOVA with Tukey's HSD *post-hoc* test) (Figures 7D–D”). *Htr3a*^+/+^ average NF200+ innervation density was 3.01 ± 0.29%, while *Htr3a*^+/−^ and *Htr3a*^−/−^ innervation densities were 6.06 ± 0.83 and 7.92 ± 0.79% respectively. The difference between *Htr3a*^+/−^ and *Htr3a*^−/−^ NF200+ innervation density measurements was not statistically significant (*p* = 0.1759, one-way ANOVA with Tukey's HSD *post-hoc* test). From our experiments on adult *Htr3a* mutant tissues, we conclude that loss of *Htr3a* gene expression has sustained effects on innervation densities beyond fetal and postnatal development, in which mutant bladder smooth muscle has an overabundance of neuronal fibers, including cholinergic, neuropeptidergic, and mechanosensory.

## Discussion

While the effects of serotonin signaling in neural development have been studied for decades, the 5-HT3A serotonin receptor has only recently been recognized as a key player in developmental mechanisms (Engel et al., 2013). These recent studies of 5-HT3A signaling in neural development have been focused on the CNS. The goal of this study was to define the expression and role of 5-HT3A in the development of urinary bladder innervation and determine whether this serotonin receptor is required for normal voiding in adult mice. Using a knockout mouse model of 5-HT3A deficiency, we demonstrate a novel role for this serotonin receptor in the development of the peripheral nervous system architecture and show that perturbing 5-HT3A signaling has lasting effects on visceral organ function in adult animals. The analysis presented has important ramifications for the potential impact of pharmacological agents taken during pregnancy that may have long-lasting effects on bladder innervation and function in later life.

Expression of 5-HT3A was first demonstrated in 14.5 dpc autonomic pelvic ganglia via *in situ* hybridization over two decades ago (Tecott et al., 1993, 1995); surprisingly, these findings have not been further investigated. Our expression analysis using the *Htr3a*-EGFP transgenic reporter allele confirms expression of 5-HT3A in fetal pelvic ganglia at stages when neurogenesis is actively occurring (Wiese et al., 2017). Moreover, our results show for the first time that the 5-HT3A receptor is maintained in adult pelvic ganglia and is expressed in the majority of adult pelvic ganglia neurons (Figure 1).

VSAs have been established as a means to quantify urinary voiding in an environment as similar to the animals' home cage environment as possible and thus eliminate many confounding factors inherent to other methods of urinary voiding assessment (Yu et al., 2014; Keil et al., 2016). Our VSA study of *Htr3a* mutant mice revealed an increase in voiding frequency in male *Htr3a* mutants compared to wild type littermates (Figure 2). This is the first report of any urinary tract dysfunction in *Htr3a* homozygous loss of function mutants. Because detection of any urinary dysfunction requires unique experimental assays and careful analysis of large animal cohorts, this aspect of the *Htr3a* knockout phenotype was most likely overlooked by prior studies of these mutants that observed the animals in their home cage environment.

Differences in voiding frequency measured in a 4-h VSA have not been previously reported for wild type post-pubescent male and female mice on a C57BL6/J background (Bjorling et al., 2015). We had anticipated that there would be no sex specific effects in our analysis of *Htr3a* mutants. Remarkably, we found that loss of the 5-HT3A receptor in *Htr3a* knockout mice differentially affects voiding patterns of males and females by VSA before the onset of reproductive maturity. The increased voiding frequency we observed in male *Htr3a* mutants suggests that 5-HT3A receptor activity normally functions in male mice to inhibit urinary voiding. It should be noted that female *Htr3a*^−/−^ animals in our VSA study exhibited a slight trending increase in urinary voiding frequency compared to wild type animals, but the increase in frequency was not statistically significant. Our observation suggests that the 5-HT3A receptor may be involved in inhibiting micturition in both males and females, but does so to a greater extent in male mice. In contrast, a pharmacological study conducted in cats demonstrated that 5-HT3A receptor signaling inhibits the micturition drive in both male and female cats (Schwen et al., 2013). A separate study examining the effects of 17alpha-estradiol and 17beta-estradiol *in vitro* found that these hormones can act as allosteric antagonists of 5-HT3A in an exogenous expression system (Wetzel et al., 1998; Oz et al., 2002), but the mechanisms by which sex hormones may alter 5-HT3A activity in regulating urinary voiding *in vivo* are unknown. Interestingly, estrogen receptors are expressed in parasympathetic pelvic ganglia neurons (Purves-Tyson et al., 2007) and sex hormones have been shown to influence pelvic ganglia neuron function (Keast, 2006), providing an avenue by which 5-HT3A may mediate urinary tract function in a sex-specific manner. Further studies focused on determining if sex hormones regulate 5-HT3A signaling *in vivo* and how this signaling contributes to sex differences in micturition circuitry would be valuable.

Because *Htr3a* is expressed in many brain regions involved in cognitive control of micturition and our study relied upon a global *Htr3a* knockout mouse line, there is the possibility that the increase in urinary frequency observed in male *Htr3a* mutants could be due to abnormal cognitive control of when and where to void. However, the anesthetized bladder cystometry assay we applied addresses this issue by specifically measuring involuntary, autonomic urinary bladder voiding (Figure 3). The finding that male *Htr3a* mutants exhibit decreased voiding efficiency is consistent with the results obtained in our VSA testing. The results of both of these experiments together suggest that 5-HT3A receptor signaling is required in males for maintaining normal urinary tract function. There are multiple aspects of LUT function that could be affected by the loss of 5-HT3A activity, including bladder detrusor contractility and its coordination with urethral sphincter relaxation (detrusor-sphincter dyssynergia). Future studies combining the use of cystometry with electromyography recordings of the external urethral sphincter would be valuable for elucidating these mechanisms in *Htr3a* mutants.

A prior study utilizing the *Htr3a*^*vs*/*vs*^ mouse model, which expresses a constitutively active hypersensitive form of the 5-HT3A receptor, also reported poor urinary bladder function in adult males (Bhattacharya et al., 2004) that were accompanied by bladder wall hyperplasia. However, in contrast to the deficits reported in the *Htr3a*^*vs*^ hypersensitive mutants, we did not find any evidence of bladder outlet obstruction or other overt LUT malformations in *Htr3a*KO mice. Our findings in concert with those of Bhattacharya et al. indicate that the control of micturition in mice is acutely sensitive to levels of 5-HT3A receptor activity and highlight the importance of interrogating gene function from multiple angles. Both the *Htr3a*^−/−^ and the *Htr3a*^*vs*/*vs*^ mutants are constitutive alleles, with the effect of the receptor variants exerted through the entire window of endogenous gene expression. Given that *Htr3a* is expressed during LUT development and into adulthood, our studies raise the intriguing question of whether 5-HT3A receptor activity is required at a specific time during the development of bladder innervation. Use of an inducible *Htr3a* mutant allele in conjunction with assays of urinary tract function will be needed to determine the developmental time point when 5-HT3A receptor activity is most critical for establishing normal voiding patterns in adults.

The pelvic ganglia in mice are unique among autonomic structures in that they are comprised of both sympathetic and parasympathetic neurons (Keast, 1995; Wanigasekara et al., 2003). Given the widespread and sustained expression of 5-HT3A in these structures, we sought to determine if *Htr3a* knockout would affect expression of autonomic neuronal subtype markers. Interestingly, we observed expression of TH is visibly up-regulated in *Htr3a* homozygous mutant fetal pelvic ganglia compared to wild type and heterozygous littermates, with a concurrent down-regulation of cholinergic marker ChAT. However, by adulthood the discernable differences in TH+ and ChAT+ cells seen at 14.5dpc were no longer apparent (Figures 4, 5). Prior work has established that TH and ChAT levels in pelvic ganglia neurons can be profoundly affected by circulating sex hormones (Melvin and Hamill, 1987; Keast, 2006); thus, it is possible that 5-HT3A receptor signaling is not required for post-pubescent TH and ChAT gene expression. Stage-specific effects of 5-HT3A knockout to discern the effects of this receptor on neurochemical profiles in the pelvic ganglia could be explored in the future with an inducible *Htr3a* mutant allele.

The 5-HT3A receptor has been previously shown to be critical for the morphological maturation of neurons in the CNS (Chameau et al., 2009; Smit-Rigter et al., 2011; Oostland et al., 2013), but whether *Htr3a* expression is required for normal peripheral neuronal morphology has not been previously investigated. In the present study, we found that loss of *Htr3a* gene expression leads to an excess of neuronal fibers innervating the smooth muscle of the bladder, indicating the requirement of this receptor for the normal maturation of neural architecture in peripheral tissue. Regardless of *Htr3a* genotype, bladder smooth muscle intensity was reduced in adult tissues compared to 18.5dpc (with exception being CGRP+ fibers, Figures 6, 7). This is likely due to the pruning of neuronal branches that normally occurs in postnatal development (Purves and Lichtman, 1980; Riccomagno and Kolodkin, 2015). The exception of CGRP+ fibers suggests that there are neuronal subtype-specific differences in the time course of neuronal process outgrowth and maturation. It is also possible that expression of CGRP in neuronal fibers within the bladder changes over postnatal development. Our prior study examining gene expression profiles in developing dorsal root ganglia indicates that CGRP expression is somewhat dynamic through development (Ritter and Southard-Smith, 2017), but additional work is needed to explore these possibilities further.

The observation that loss of one or both copies of the *Htr3a* gene leads to an increase in bladder smooth muscle innervation density of all four neuronal markers examined suggests that 5-HT3A may act to either inhibit neuronal fiber outgrowth, or may possibly be involved in the process of fiber pruning in postnatal development. Other research groups have reported similar effects of 5-HT3A loss; the apical dendrites of layer II/III cortical pyramidal neurons in the brain exhibit significantly more complex branching morphology in *Htr3a*^−/−^ mice compared to wild type littermates (Chameau et al., 2009; Smit-Rigter et al., 2011). However, other groups have reported contradictory results from *in vitro* studies of this receptor—in PC12 cells, 5-HT3A receptor activation promotes neurite outgrowth via increased calcium flux (Homma et al., 2006). Interestingly, the study by Bhattacharya et al. using the hyperactive *Htr3a*^*vs*^ variant described reduced nerve fiber density in bladder smooth muscle whereas our analysis looking at loss of *Htr3a* function identified increased innervation. Together, these findings suggest that the density of bladder innervation is dependent on the levels of 5-HT3A receptor activity. In light of these results, future studies are needed to identify the molecular factors downstream of 5-HT3A activation that govern neuronal architecture in peripheral neurons as a pathway to defining mechanisms that control bladder innervation.

Our results collectively have important implications for human neural development. The changes in adult male voiding function that derive from loss of 5-HT3A in mice may occur as a result of deficient 5-HT signaling in the fetus, the adult, or both. While mouse studies cannot be directly translated to humans, the results of altered 5-HT3A receptor signaling that we describe suggest more thorough investigation focused on the outcomes of pharmacological intervention at this receptor during gestation is warranted. Ondansetron, a potent 5-HT3A receptor antagonist, is commonly prescribed to pregnant women to treat severe nausea (Mcparlin et al., 2016; Taylor et al., 2017). Additionally, the selective serotonin reuptake inhibitor (SSRI) fluoxetine, which is also prescribed frequently to pregnant women for the treatment of depression, has been shown by other groups to interact with 5-HT3A (Eisensamer et al., 2003; Smit-Rigter et al., 2012). Overall, the current study highlights the importance of 5-HT3A receptor signaling in adult urinary bladder function and demonstrates a novel role for this receptor in autonomic and sensory nervous system development.

## Author contributions

KER: designed research, performed research, analyzed data, and wrote the paper; ZW: performed research and analyzed data; CMV: designed research and analyzed data; DEB: designed research and analyzed data. EMS-S: designed research, analyzed data, and wrote the paper.

### Conflict of interest statement

The authors declare that the research was conducted in the absence of any commercial or financial relationships that could be construed as a potential conflict of interest.
