# Supplementary material for: Serotonin Receptor 5-HT3A Affects Development of Bladder Innervation and Urinary Bladder Function
Source: Front Neurosci. 2017 Dec 12;11:690. doi: 10.3389/fnins.2017.00690 (PMC5732969; doi:10.3389/fnins.2017.00690)
Supplement: Supplementary file 1 [file Image1.PDF]

Supplementary Material

Serotonin receptor 5-HT3A affects development of bladder innervation and urinary bladder function

K. Elaine Ritter, Zunyi Wang, Chad M. Vezina, Dale E. Bjorling, E. Michelle Southard-Smith*

*** Correspondence:** E. Michelle Southard-Smith: michelle.southard-smith@vanderbilt.edu

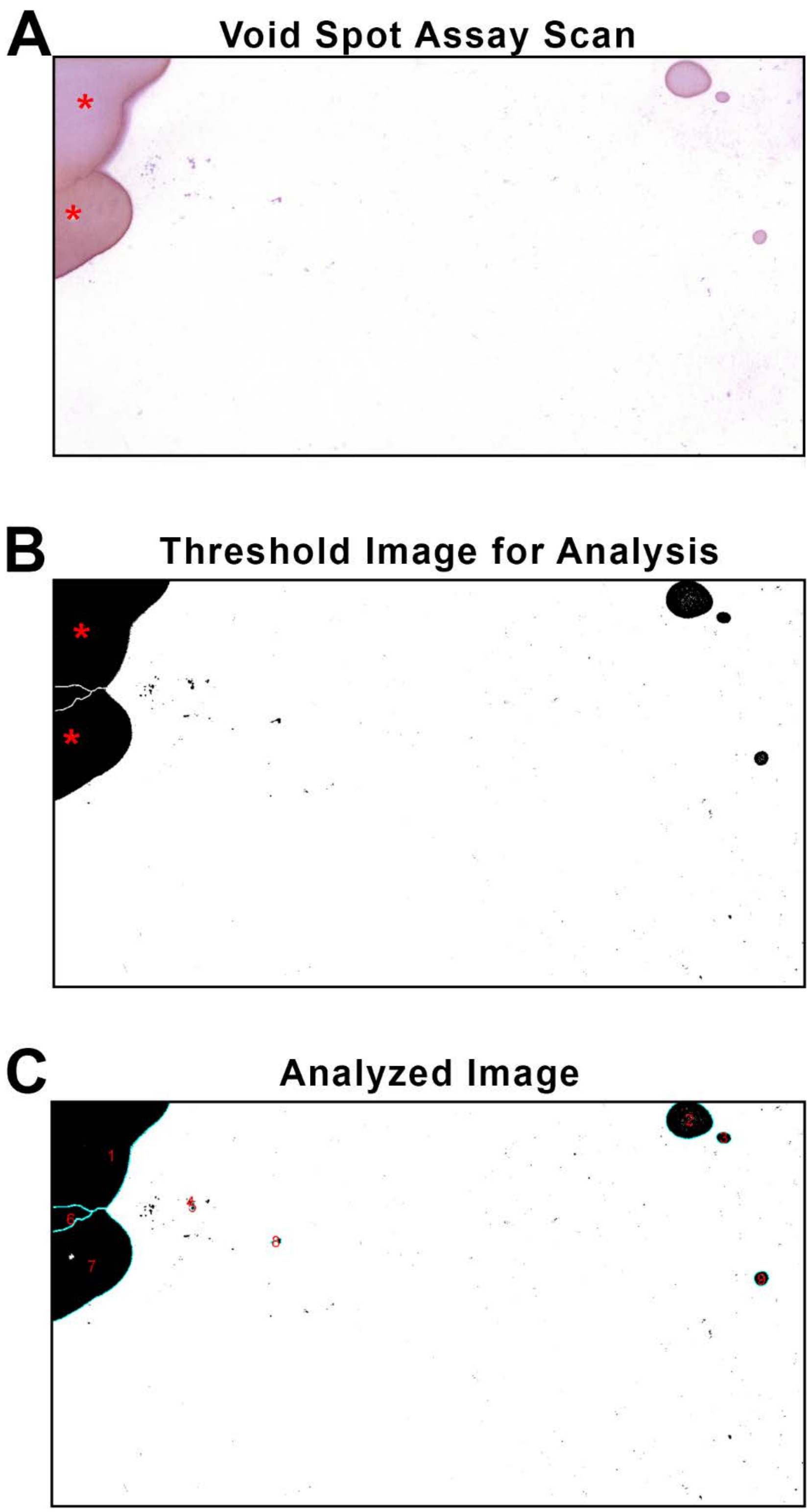

Supplementary Figure 1. Analysis method for quantifying overlapping void spots in the void spot assay. (A) Scan of Ninhydrin-stained void spot assay paper containing two overlapping void

spots (denoted with red asterisks). (B) Image of void spot assay paper shown in A that has been thresholded for analysis. White lines were drawn over the borders between the two overlapping void spots (denoted with red asterisks) so they could be separately counted. (C) Analyzed image showing the Ninhydrin-stained spots that were measured in the analysis. Counted void spots are enumerated in red and outlined in cyan. The overlapping region (denoted as “6” in this figure) was counted twice for void surface area, but was not included in the total void spot number count. This method of quantification allows a more accurate measurement of void surface area. Note that very small spots ($<0.02cm^2$) were excluded from the analysis, as these extremely small spots are likely debri or background staining.
